# The regulation of ISG20 expression on SARS-CoV-2 infection in cancer patients and healthy individuals

**DOI:** 10.3389/fimmu.2022.958898

**Published:** 2022-09-13

**Authors:** Jingliang Cheng, Jiewen Fu, Qi Tan, Zhiying Liu, Kan Guo, Lianmei Zhang, Jiayue He, Baixu Zhou, Xiaoyan Liu, Dabing Li, Junjiang Fu

**Affiliations:** ^1^ Key Laboratory of Epigenetics and Oncology, The Research Center for Preclinical Medicine, Southwest Medical University, Luzhou, China; ^2^ Department of Pathology, The Affiliated Huaian No. 1 People’s Hospital of Nanjing Medical University, Huai’an, China; ^3^ Department of Gynecology and Obstetrics, Guangdong Women and Children Hospital, Guangzhou, China; ^4^ Basic Medical School, Southwest Medical University, Luzhou, China

**Keywords:** ISG20 expression, cancer, SARS-CoV-2, cordycepin (CD), N6, N6-dimethyladenosine (m 6 2A)

## Abstract

ISG20 inhibits viruses such as SARS-CoV-2 invasion; however, details of its expression and regulation with viral susceptibility remain to be elucidated. The present study analyzed ISG20 expression, isoform information, survival rate, methylation patterns, immune cell infiltration, and COVID-19 outcomes in healthy and cancerous individuals. Cordycepin (CD) and N6, N6-dimethyladenosine (m^6^_2_A) were used to treat cancer cells for ISG20 expression. We revealed that *ISG20* mRNA expression was primarily located in the bone marrow and lymphoid tissues. Interestingly, its expression was significantly increased in 11 different types of cancer, indicating that cancer patients may be less vulnerable to SARS-CoV-2 infection. Among them, higher expression of ISG20 was associated with a long OS in CESC and SKCM, suggesting that ISG20 may be a good marker for both viral prevention and cancer progress. *ISG20* promoter methylation was significantly lower in BLCA, READ, and THCA tumor tissues than in the matched normal tissues, while higher in BRCA, LUSC, KIRC, and PAAD. Hypermethylation of *ISG20* in KIRC and PAAD tumor tissues was correlated with higher expression of *ISG20*, suggesting that methylation of *ISG20* may not underlie its overexpression. Furthermore, ISG20 expression was significantly correlated with immune infiltration levels, including immune lymphocytes, chemokine, receptors, immunoinhibitors, immunostimulators, and MHC molecules in pan-cancer. STAD exhibited the highest degree of *ISG20* mutations; the median progression-free survival time in months for the unaltered group was 61.84, while it was 81.01 in the mutant group. Isoforms ISG20-001 and ISG20−009 showed the same RNase_T domain structure, demonstrating the functional roles in tumorigenesis and SARS-CoV-2 invasion inhibition in cancer patients. Moreover, CD and m^6^_2_A increase ISG20 expression in various cancer cell lines, implying the antiviral/anti-SARS-CoV-2 therapeutic potential. Altogether, this study highlighted the value of combating cancer by targeting ISG20 during the COVID-19 pandemic, and small molecules extracted from traditional Chinese medicines, such as CD, may have potential as anti-SARS-CoV-2 and anticancer agents by promoting ISG20 expression.

## 1 Introduction

Interferon stimulated exonuclease gene 20 (ISG20, OMIM: 604533) aliases HEM45, CD25, Promyelocytic leukemia nuclear body-associated protein ISG20, interferon-stimulated exonuclease gene 20 kDa, interferon-stimulated gene 20 KDa protein, estrogen-regulated transcript 45 protein, and EC 3.1.13.1. ISG20 cytogenetic locates on chromosome 15q26.1 and genomic coordinates (GRCh38) between 15:88,635,631 and 88,656,4820 was first isolated by Gongora et al. in 1997 as a cDNA encoding an interferon-induced protein, called ISG20, by screening an IFN-treated Daudi cell cDNA library (1). Pentecost (1998) identified a cDNA that encoded a 181-amino acid protein with a predicted molecular weight of 20,363 Da; the expression of *ISG20* mRNA was increased in response to estrogen in estrogen receptor-expressing cells in the presence of cycloheximide (2).

ISG20 is predicted to exhibit a broad spectrum of antiviral activity, including hepatitis A virus (HAV), hepatitis B virus (HBV), hepatitis C virus (HCV), Influenza A virus (IAV), and yellow fever virus (YFV) in an exonuclease-dependent manner, through the degradation of viral RNA as a 3’-5’-exoribonuclease (3, 4). Additional antiviral mechanisms by ISG20 include translational inhibition of viral RNA and non-self RNAs and degradation of deaminated viral DNA (3, 5–7). ISG20 has also been reported to inhibit the replication of bluetongue virus (BTV) in ovine (8) and to inhibit the proliferation of pseudorabies virus (PRV) (9, 10). Furthermore, a recent study suggested that ISG20 can degrade SARS-CoV-2 (severe acute respiratory syndrome coronavirus 2) sub-replicon RNA through exonuclease activity (11). The SARS-CoV-2 is the pathogen underlying the current COVID-19 (coronavirus disease 2019) pandemic, leading to more than 596 million positive cases and 6 million deaths worldwide (https://coronavirus.jhu.edu/).

Similarly, ISG20 acts as a SARS-CoV-2 RNase and is critical in inhibiting the SARS-CoV-2 replicon in host cells. Therefore, the expression and distribution of ISG20 may explain the differences in COVID-19 severity after the SARS-CoV-2 invasion. Cellular and humoral immunity participate in the prevention of viral invasion, and the pathological process of COVID-19 is likely correlated with the dysregulation of the immune response, particularly of T cells. Targeting ISG20 may thus be a potential therapeutic strategy for managing SARS-CoV-2 infection.

A large body of evidence has indicated the effect of COVID-19 on the clinical outcomes of cancer patients. According to cohort studies of COVID-19 on the Cancer Consortium and systematic reviews, patients with cancer and COVID-19 exhibit increased mortality rates (12–15). Thus, increased attention should be paid to patients with cancer during the COVID-19 pandemic.

Herein, we performed comprehensive and integrative profiling of ISG20 expression in healthy individuals and patients using a pan-cancer dataset using genomic, transcriptomic, and epigenomic data. The relationships between the expression of ISG20 and immune cell infiltration were investigated. These results may highlight the significance of SARS-CoV-2 infection in patients with different cancer types and the potential therapeutic value of using small molecules such as cordycepin (CD) and N6, N6-dimethyladenosine (m^6^_2_A) in managing SARS-CoV-2 infection.

## 2 Materials and methods

### 2.1 Online databases

ISG20 homologs in humans from GenBank (Protein: NP_001290162.2, Gene: NM_001303233.2) and others were obtained from NCBI (National Center for Biotechnology Information) (https://www.ncbi.nlm.nih.gov/homologene/31081) (16, 17). Data on gene and protein expression levels of ISG20 in the normal and cancerous tissues (https://www.proteinatlas.org/ENSG00000172183-ISG20/tissue), in different types of immune cells (RNA) (https://www.proteinatlas.org/ENSG00000172183-ISG20/immune+cell), in single cells (https://www.proteinatlas.org/ENSG00000172183-ISG20/single+cell+type), and brain tissues (https://www.proteinatlas.org/ENSG00000172183-ISG20/brain) were obtained from the Human Protein Atlas (HPA) (18, 19). *ISG20* expression in different types of cancer tissues and the corresponding normal tissues, isoform, distribution, and domain structures were analyzed using GEPIA 2 (gene expression profiling interactive analysis 2) (http://gepia2.cancer-pku.cn/#analysis) and (http://gepia2.cancer-pku.cn/#isoform) (20, 21). DNA methylation analysis of the *ISG20* promoter was performed using DNMIVD (DNA methylation interactive visualization database) (http://119.3.41.228/dnmivd/query_gene/?cancer=pancancer&gene=ISG20) (22). Data on *ISG20* mutations were obtained from cBioPortal for cancer genomics (https://www.cbioportal.org/results/cancerTypesSummary?case_set_id=all&gene_list=ISG20&cancer_study_list=5c8a7d55e4b046111fee2296) (23). Survival analysis of *ISG20* expressions was performed using GEPIA 2, DNMIVD, and cBioPortal. Analysis of the relationships between the abundance of tumor-infiltrating lymphocytes (TILs) and expression was performed using TISIDB (an integrated repository portal for tumor-immune system interactions) (http://cis.hku.hk/TISIDB/browse.php?gene=ISG20) (24).

### 2.2 Immunohistochemistry analysis

Immunohistochemistry (IHC) in formalin-fixed, paraffin-embedded breast cancer tissue sections from Chinese patients was performed as described previously (17, 25–27). The ISG20 antibody (C-12, cat #: sc-514979) for IHC and western blotting was purchased from Santa Cruz Biotechnology, Inc., USA. For details, 5μm deparaffinized and rehydrated sections were incubated in 10 µM sodium citrate buffer at 95°C for 12 min for antigen retrieval, and treated with 3% hydrogen peroxide. Then blocking with 5% bovine serum albumin (BSA). Primary ISG20 antibody (1:50 dilution) was applied overnight and then incubated with appropriate biotin-conjugated secondary antibodies (SP-9000, ZSGB-Bio, CN) for 60 min at 25°C. Immunostaining signals were visualized by the Streptavidin-conjugated horseradish peroxidase (HRP) and 3,3-diaminobenzidine (DAB) (ZLI-9017, ZSGB-Bio, CN). Slides were counterstained with hematoxylin, dehydrated, and mounted.

### 2.3 Cell culture

Cancer cell lines A549, H1975, HepG2, 22RV1, PC3, BT549, MDA-MB-231, and HeLa were obtained from ATCC (American Type Culture Collection), and cultured in DMEM or RPM1640 supplemented with 10% serum and 1% penicillin-streptomycin (Gibco; Thermo Fisher Scientific, Inc.) in 12-well plates. CD (Cat #: A0682) was obtained from Chengdu Must Bio-Technology Co. Ltd (Chengdu, Sichuan, China), m^6^_2_A (CAS #: 2620-62-4) from BOC Sciences (Shirley, NY, USA), and uridine-5’-monophosphate (UMP, CAS #: 58-97-9) from Shanghai Aladdin Biochemical Technology company (Shanghai, China). Total RNA and protein were extracted after UMP, CD, or m^6^_2_A treatment with the indicated concentrations for 24 h. The cells were lysed using EBC buffer (20 mM Tris-HCl, pH 8.0, 125 mM NaCl, 2 mM EDTA, and 0.5% NP-40) supplemented with a protease and phosphatase inhibitor cocktail. The harvested protein was stored at -20°C until required.

### 2.4 Western blotting

SDS-PAGE was used for western blotting. After electrophoresis at 100v for 100 min, the proteins were transferred to membranes at 100v for approximately 90 min. Then the membranes were blocked with fresh 5% fat-free milk at room temperature for 2 h. Primary antibodies against ISG20, β-actin, or HSP90 were incubated in the fresh 2% fat-free milk at 4°C overnight. The following day, membranes were washed three times with TBST (Tris-buffered saline containing 0.1% Tween20) for 15 min each time, and the blots were incubated with an anti-mouse HRP secondary antibody (1:5000 dilution) in 2% fat-free milk for a further 2 h. Subsequently, the membranes were three times as above. The protocol for western blot in breast cancer tissues and its matched healthy tissues from Chinese breast cancer patients was described previously (25, 26). All experiments were repeated three times.

### 2.5 Semi-quantitative reverse transcription-polymerase chain reaction

The harvested total RNA was reverse transcribed into cDNA. The sequences of the primers targeting *ISG20* (NM_001303233.2) were: RT-ISG20-5: 5’-cttccaggcactgaaagagg-3’ (forward primer), RT-ISG20-3: 5’-aagccgaaagcctctagtcc-3’ (reverse primer). The expected product size was 309 bp. These primers would expect to detect isoforms ISG20-001 and ISG20−009, two main isoforms for ISG20 in **Figure 5C**. *ACTB* was used as the internal control. The sequences of the primers ACTB were: RT-ACTB-5: 5’-CTCTTCCAGCCTTCCTTCCT-3’ (forward primer), RT-ACTB-3:5’-CACCTTCACCGTTCCAGTTT-3’ (reverse primer). The expected product size was 510 bp. Semi-quantitative RT-PCR was performed as described previously (28). All experiments were repeated three times.

### 2.6 Statistical analysis

To compare the expression of ISG20 in pan-cancer and in the matched healthy tissues, |log2FC| values were used and log-rank *P*<0.05 was considered statistically significant. For comparison of the methylation of the ISG20 promoter region in cancer tissues and the corresponding healthy tissues, a student’s t-test was used, and *P-value <*0.05 was considered significant.

## 3 Results

### 3.1 Expression of ISG20 in normal tissues

**Figure 1:**
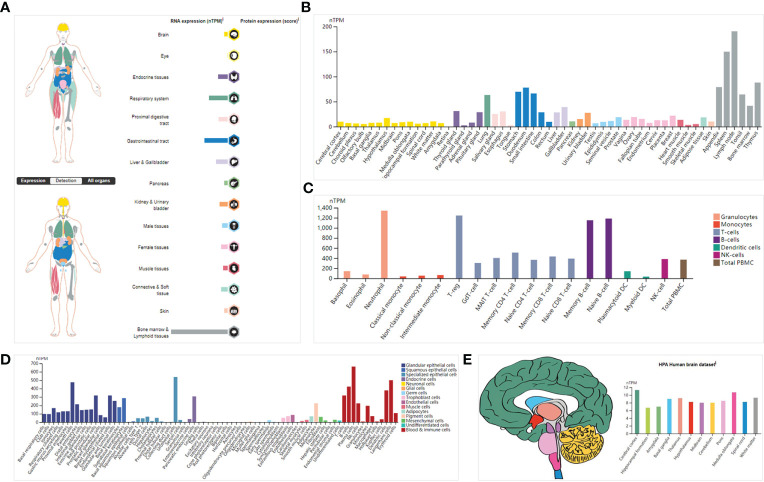
The expression of ISG20 in healthy individuals. **(A)** Overview of ISG20 expression and distribution across different types of healthy human tissues. **(B)** mRNA expression of ISG20 in healthy human tissues from the consensus data set. **(C)** mRNA expression of ISG20 in human tissues in different types of immune cells. **(D)** mRNA expression of ISG20 in human tissues in different types of single cells. **(E)** mRNA expression of ISG20 in human brain tissues.

The transcriptional data on *ISG20* expression in human organs and tissues is presented in **Figure 1A**; *ISG20* mRNA was mainly located in the bone marrow and lymphoid tissues, followed by the gastrointestinal tract, respiratory system, liver and gallbladder, endocrine tissues, and kidney and urinary bladder, with no expression in the eyes. High expression of *ISG20* in the respiratory system (lung, 63.3nTPM) demonstrated its antiviral role in the lungs. The expression of *ISG20* mRNA was further validated in the consensus data set (**Figure 1B**). In agreement with the above results, the top nine tissues/organs for *ISG20* mRNA expressions in this consensus dataset were the lymph node, spleen, thymus, appendix, and tonsils (they are bone marrow and lymphoid tissues), stomach, duodenum, and small intestine (they are gastrointestinal tract), and lungs (**Figure 1B**). Then, the mRNA expression levels of *ISG20* were examined in human tissues of immune cells, single cell types, and the brain. The results of 18 immune cell types and total peripheral blood mononuclear cells (PBMCs) indicated that *ISG20* mRNA expression was very high in neutrophils, T-regs, memory B-cells, and naive B-cells (all >1,150 nPTM) (**Figure 1C**). The *ISG20* mRNA expression in single-cell-type specificity indicated it was predominantly expressed in the plasma cells (663.4 nTPM), Langerhans cells (500.2 nTPM), B-cells (424.2 nTPM), dendritic cells (378.8 nTPM), T-cells (318.4 nTPM), urothelial cells (538.6 nTPM), and paneth cells (476.1 nTPM) (**Figure 1D**). The mRNA expression levels of *ISG20* in the brain were very low but remained detectable, with the highest levels observed in the cerebral cortex (11.3 nPTM) (**Figure 1E**). The *ISG20* expression was unavailable (NA) in human blood cells from HPA.

**Figure 2:**
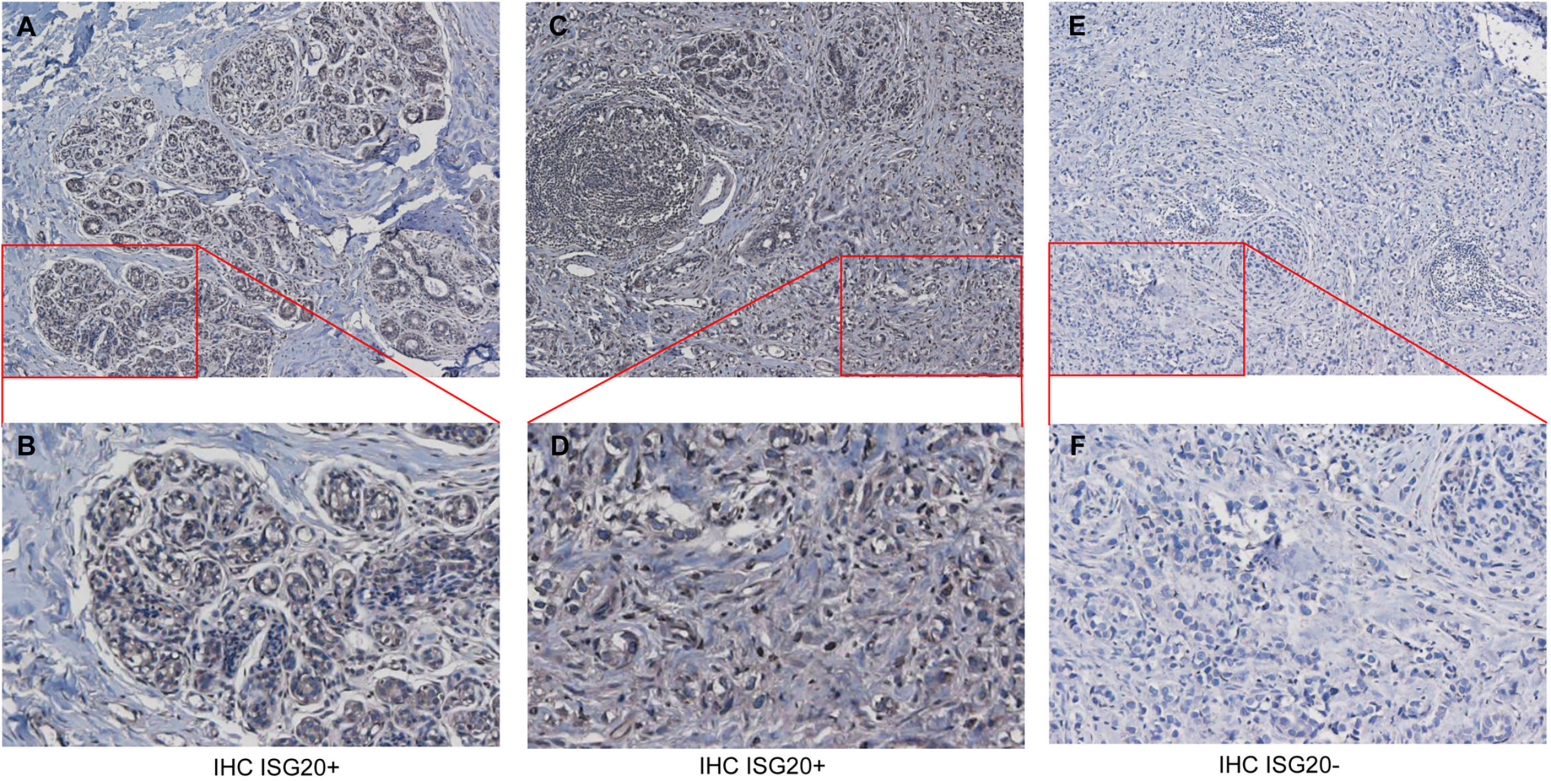
Immunohistochemistry (IHC) analysis of ISG20 expression healthy and cancer tissues from breast cancer patients. **(A, B)** IHC analysis of ISG20 in breast tissues. **(C, D)** IHC analysis of ISG20 in breast cancer tissues. **(E, F)** Control IHC of breast tissues without antibody. Panels **(B, D)** show enlarged insets from **(A, C)**, respectively.

Next, we conducted IHC of breast cancer tissues; representative results are shown in **Figures 2A–F**. ISG20 staining showed high expression in the cytoplasm and membranes in the breast tissues (**Figures 2A, B**) and breast cancer tissues (**Figures 2, D**). ISG20 was primarily located in the cytoplasm and membrane (highest in the cytoplasm), indicating its role in viral prevention. As a control, we also showed IHC images of breast tissues and breast cancer tissues without antibodies, respectively, in **Figures 2E, F**.

### 3.2 *ISG20* expression is increased in cancer tissues compared with the corresponding normal tissues

**Figure 3:**
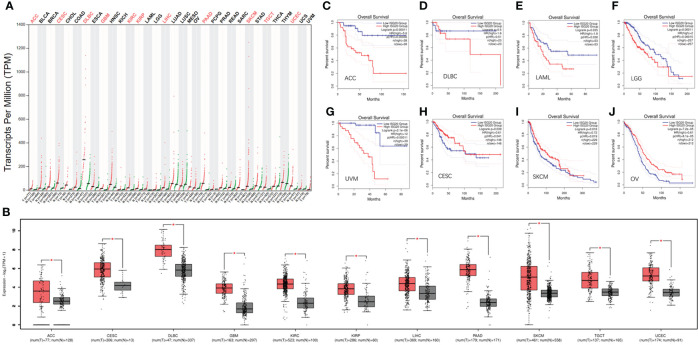
The expression of ISG20 in pan-cancer and the respectively matched healthy tissues. **(A)** The expression of ISG20 in different types of cancer and the corresponding normal tissues. **(B)** The expression of ISG20 was significantly higher in the cancer tissues compared with the corresponding normal tissues. **(C–J)**. Survival and ISG20 expression in ACC, DLBC, LAML, LGG, UVM, CESC, SKCM, and OV patients. |log2FC| values were used, and log-rank *P*<0.05 was considered statistically significant. *indicates the significant difference.

Increasing evidence has shown that cancer patients are more vulnerable to SARS-Cov-2. As an enigmatic antiviral factor, it is important to know the expression levels of ISG20 in cancer tissues compared with corresponding healthy tissues. Surprisingly, *ISG20* mRNA expression was significantly increased in eleven types of cancer, including ACC (adrenocortical carcinoma), CESC (cervical squamous cell carcinoma and endocervical), DLBC (lymphoid neoplasm diffuse large B-cell lymphoma), GBM (glioblastoma multiforme), KIRC (Kidney renal clear cell carcinoma), LIHC (liver hepatocellular carcinoma), KIRP (kidney renal papillary cell carcinoma), PAAD (pancreatic adenocarcinoma), SKCM (skin cutaneous melanoma), TGCT (testicular germ cell tumors), and UCEC (uterine corpus endometrial carcinoma) (**Figures 3A, B**) compared with the matching normal tissue. Thus, high ISG20 expression in cancer may prevent viral invasion in these cancer patients.

To further validate the expression results, samples of breast cancer tissues and their matched healthy tissues were selected for collection and western blot since ISG20 levels are increased even though not significantly (**Supplementary Figure 1A**). It is also easy for us to collect breast tumor tissues. After western blot and the results were presented in **Supplementary Figure 1B**, ISG20 protein levels were increased significantly in 6 of 10 samples/patients (60%) of cancer tissues compared with the matched healthy tissues (**Supplementary Figure 1B**). These results validated the mRNA results from the TCGA database for BRCA (breast invasive carcinoma) patients.

### 3.3 The prognostic value of *ISG20* in pan-cancer

Further exploration of the prognostic value of *ISG20* revealed that higher expression was associated with a shorter OS in ACC, DLBC, LAML (acute myeloid leukemia), LGG (lower grade glioma), and UVM (uveal melanoma) (**Figures 3C–G**), but with a long OS in CESC, OV (ovarian serous cystadenocarcinoma), and SKCM (**Figures 3H–J**). It was reported that ISG20 overexpression suppressed the proliferation, migration, and invasion *in vitro* and the growth of xenograft tumors *in vivo* in ovarian cancer (29), and it may be associated with a long OS in OV patients.

High expression of *ISG20* in CESC and SKCM was associated with a longer OS, suggesting that ISG20 may be a good marker. However, high expression of ISG20 in ACC and DLBC was associated with a shorter OS, suggesting that ISG20 may be a marker of unfavorable outcomes in these types of cancer. Together, ISG20 may serve as a double-edged sword in viral prevention and cancer progression in certain types of cancer.

### 3.4 Methylation of the *ISG20* promoter region in cancer and the matched normal tissues

**Figure 4:**
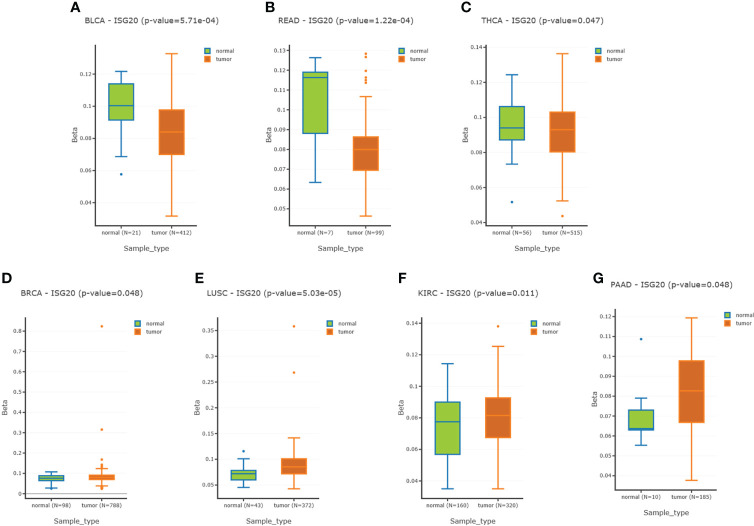
Methylation of the ISG20 promoter region in cancer tissues and the corresponding healthy tissues. **(A–C)**. Methylation of the ISG20 promoter region in cancer tissues was significantly lower than in the corresponding healthy tissues for BLCA, READ, and THCA, respectively. **(D–G)**. Methylation of the ISG20 promoter region in cancer tissues was significantly higher than in the corresponding healthy tissues for BRCA, LUSC, KIRC, and PAAD, respectively. The student’s t-test was used and *P-value <*0.05 was considered significant.

DNA methylation can regulate gene expression. We’d like to know whether ISG20 expression changes are due to methylation modification. By analyzing the DNMIVD database, we found that *ISG20* promoter methylation was significantly lower in BLCA, READ, and THCA tumor tissues compared to the matching normal tissue (**Figures 4A–C**), while higher in BRCA, LUSC, KIRC, and PAAD (**Figures 4D–G**). Hypermethylation of *ISG20* in KIRC and PAAD tumor tissues was correlated with the higher expression, suggesting that methylation of *ISG20* may not be the cause of overexpression. Thus, other mechanisms may be involved in regulating *ISG20* expression.

### 3.5 Expression distribution, utilization, and structure of *ISG20* in pan-cancer, and conservation across different species

**Figure 5:**
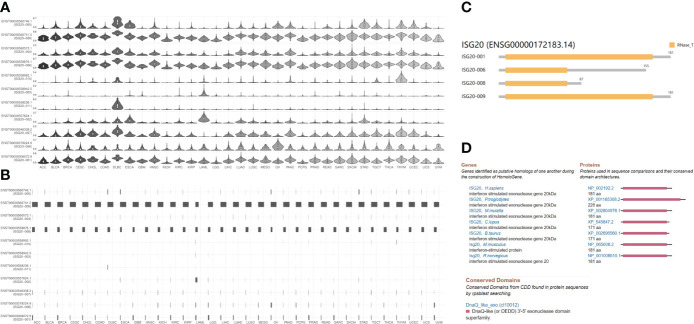
ISG20 isoform expression distribution, utilization, structure in pan-cancer, and conservation of ISG20 across different species. **(A)** The expression profiles of the ISG20 isoforms (violin plot). **(B)** Utilization profiles of the ISG20 isoforms (bar plot). **(C)** Structure of the ISG20 isoforms in pan-cancer. Information on 7 isoforms is missing; specifically, ENST00000546338.2, ENST00000557824.1, ENST00000558236.1, ENST00000558942.5, ENST00000558992.1, ENST00000560573.1, and ENST00000560746.1. **(D)** Conservation of ISG20 across different species.

Different *ACE2* isoforms have differential roles in host susceptibility to SARS-CoV-2 entry (30, 31). We analyzed *ISG20* isoform prevalence and structures in pan-cancer and found 11 isoforms that exhibited differential expression levels (**Figure 5A**). Except for very low or no expression of isoforms ENST00000558992.1 (ISG20−010), ENST00000558942.5 (ISG20−003), and ENST00000558236.1 (ISG20−011), the remaining eight *ISG20* isoforms were detectable in all cancers.

The utilization of isoform ENST00000560741.5 (ISG20−009) was the highest across all 31 cancer types, followed by ENST00000559876.1 (ISG20−006); others showed very low or no utilization (**Figure 5B**). The genomic structures of *ISG20* isoforms in pan-cancer are shown in **Figure 5C**. The isoforms ENST00000306072.9 (ISG20-001) and ENST00000560741.5 (ISG20−009) showed the same structure consisting of 181 amino acids with an RNase_T domain as reported previously; isoforms ENST00000559876.1 (ISG20-006) with 155 amino acids and ENST00000379224.9 (ISG20-008) with 87 amino acids, both possessed a truncated RNase_T domain (**Figure 5C**), demonstrating the functional role of ISG20-001 and ISG20−009 in tumorigenesis and SARS-CoV-2 invasion inhibition in cancer patients.

In addition, the ISG20 protein showed a highly conserved sequence across different species, including humans, chimpanzee, Rhesus monkey, cows, dog, mice, and rats (**Figure 5D**), suggesting that ISG20 may possess a similar potential function in inhibiting viral infection in other species (8). Indeed, ISG20 was also reported to inhibit the bluetongue virus (BTV) replication in sheep (8).

### 3.6 Mutation profiles of *ISG20* in pan-cancer

**Figure 6:**
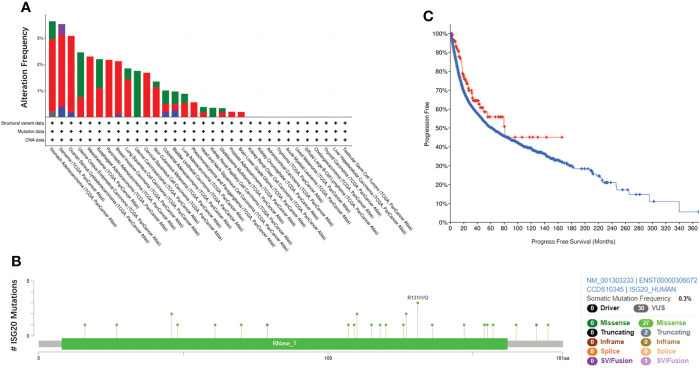
ISG20 mutations in pan-cancer. **(A)** Overview of ISG20 mutations in pan-cancer. Different colors represent different types of mutations. **(B)** ISG20 mutation hot spots in pan-cancer. **(C)** Correlation of progression-free survival between the ISG20 mutated group (red) and unaltered group (blue) of pan-cancer.

Gene mutations can cause cancer, recurrence, and/or therapeutic resistance. By analyzing the *ISG20* mutation profile in 32 types of cancer based on data obtained from TCGA, we found that STAD (stomach adenocarcinoma) had the highest mutational frequency, with 3.64% of 440 cases possessing a mutation, followed by SARC (3.53% of 255 cases), whereas BLGG (brain lower grade glioma) had the lowest frequency of mutations (0.19% of 514 cases) (**Figure ​6A**). No *ISG20* mutations were found in the other 11 types of cancer shown in **Figure 6A**. The detailed landscape of mutations shows the presence of missense mutations, truncations, and SV/fusions in the ISG20 gene, with missense mutations being the most common (**Figure 6B**).

To further explore the resulting prognostic value, we analyzed the survival correlation between *ISG20* mutant groups and unaltered groups in cancer. However, no significant difference was observed (*P*=0.0679), and the median number of months of progression-free survival for the unaltered group was 61.84 months (56.05-66.11, 95% CI), while in the mutant groups, it increased to 81.01 months (48.89-NA, 95% CI) (**Figure ​6C**).

### 3.7 Association analysis of *ISG20* expression with the tumor-immune system in pan-cancer

**Figure 7:**
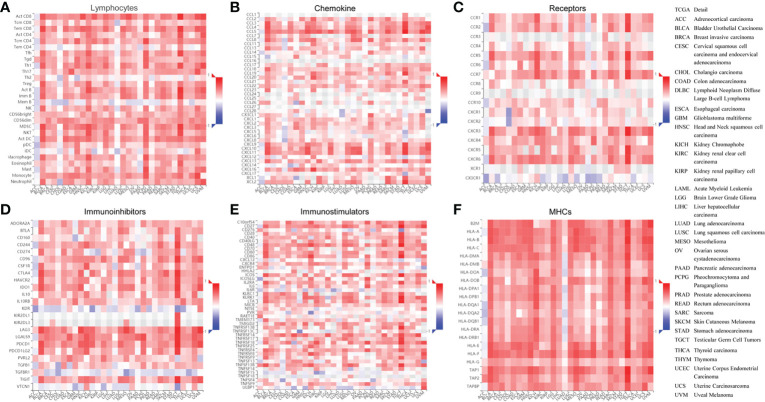
Correlation between ISG20 expressions and the tumor-immune system in pan-cancer. The correlation between ISG20 expression and **(A)** lymphocytes, **(B)** chemokines, **(C)** receptors, **(D)** immunoinhibitors, **(E)** immunostimulators, and **(F)** MHCs pan-cancer. The Y axes indicate human immune molecules, and the X axes show the human cancer type. MHC, major histocompatibility complex. The rho value was used to show the Spearman correlations with the range of -1(in blue) to 1 (in red), from the most reverse correlations to the most positive correlations of the gene expressions in the heatmaps. The right panel shows the full terms for all cancers included.

Due to the indispensability of antiviral processes and anti-tumor responses of the immune system, the correlation between *ISG20* expression and immune infiltration levels in pan-cancer was analyzed in the TISDB database. We also found significant correlations between *ISG20* expression and immune lymphocytes, chemokines, receptors, immunoinhibitors, immunostimulators, and major histocompatibility complex (MHC) molecules in almost cancer types assessed (**Figures 7A–F**).

### 3.8 CD increases ISG20 expression in various cancer cell lines

Some small molecules or natural active components can affect gene expression. We wanted to determine whether small molecules or natural components targeted ISG20 expression. To do this, we first used the DrugBank database and revealed that UMP (DB03685) might target ISG20 ​(**Table 1**; **Supplementary Figures 2A, B**). Then, several cancer-cell lines were cultured and treated with 0, 10, 20, or 40 µM UMP for 24 h, and cells were collected for RNA extraction and RT-PCR. However, the results showed that UMP did not affect *ISG20* mRNA expression in A549 lung cancer cells, HeLa cervical cancer cells, 22RV1 and PC3 prostate cancer cells, and MDA-MB-231 and BT549 breast cancer cells ​(**Supplementary Figures 2C–H**).

**Table 1:** Drugs predicted to target ISG20.

Drug ID	Name	Drug type	Predicted targets	Target no.
DB03685	Uridine monophosphate	Small Molecule	B4GALT1, GLT6D1, ISG20, LSM6, UCKL1	5

**Figure 8:**
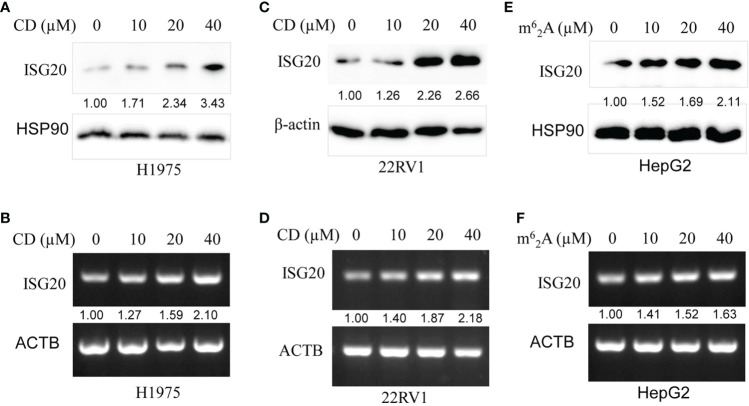
Cordycepin (CD) and N6, N6-dimethyladenosine (m^6^_2_A) increase ISG20 expression in various cancer cell lines. **(A, B)** CD increases ISG20 expression in the H1975 lung cancer cell line. **(C, D)** CD increases ISG20 expression in the 22RV1 prostate cancer cell line. **(E, F)** m^6^a_2_A increase ISG20 expression in the HepG2 live cancer cell line.

Then, CD, a nucleoside derivative, was applied to determine the effect on ISG20 expression in the cancer cell lines. The results showed that CD increased ISG20 expression at both the protein and mRNA level in a dose-dependent manner in the H1975 lung cancer-cell line ​(**Figures 8A, B**) and 22RV1 prostate cancer-cell line ​(**Figures 8C, D**).

### 3.9 m^6^_2_A increases ISG20 expression in HepG2 cancer cells

The effects of m^6^_2_A, another nucleoside derivative, on ISG20 expression in cancer cell lines were also determined. Our results showed that m^6^_2_A increased ISG20 expression at both the protein and mRNA level in a dose-dependent manner in the HepG2 liver cancer cell line ​(**Figures 8E, F**).

Altogether, both nucleoside derivatives, CD and m^6^_2_A, are predicted to exhibit antiviral/anti-SARS-CoV-2 therapeutic potential by increasing ISG20 expression.

## 4 Discussion

In this study, we found that the *ISG20* mRNA was primarily located in the bone marrow and lymphoid tissues; interestingly, the *ISG20* mRNA expression levels were significantly increased in 11 different types of cancer, including ACC, CESC, DLBC, GBM, KIRC, KIRP, LIHC, PAAD, SKCM, TGCT, and UCEC; and no decreases were observed in any type of cancer. Among these, higher expression of ISG20 was associated with a longer OS in CESC and SKCM, suggesting that ISG20 may be a good marker in both viral prevention and cancer progression in patients with these types of cancer. Unlike other receptors, such as ACE2, TMPRSS4, and CTSL, increased ISG20 expression may prevent viral invasion in these types of cancer. DNA methylation is known to affect gene expression, and we found that ISG20 promoter methylation was significantly lower in BLCA, READ, and THCA tumor tissues compared with those in the matched normal tissues, while higher in BRCA, LUSC, KIRC, and PAAD. Hypermethylation of *ISG20* in KIRC and PAAD tumor tissues was correlated with the higher expression, suggesting that methylation of *ISG20* may not underlie the increase in its expression; thus, other mechanisms may be involved in regulating *ISG20* overexpression. Interestingly, both CD and m^6^_2_A increase ISG20 expression in various cancer cell lines, even though it is unknown whether CD and m^6^_2_A regulate ISG20 expression by modification of DNA methylation patterns. Due to the indispensability of antiviral processes and anti-tumor responses in the immune system, the correlation between ISG20 expression and immune infiltration levels of pan-cancer was analyzed, and we revealed significant correlations between *ISG20* expression and immune lymphocytes, chemokine, receptors, immunoinhibitors, immunostimulators, and MHC molecules in all cancer types, highlighting a potential antiviral/anti-SARS-CoV-2 role.

Certain small molecules or natural active components can affect gene expression. We first performed DrugBank database searches and revealed ISG20 as a UMP target. UMP was demonstrated to possess an anti-fibrillatory effect by activating energy metabolism (32). Unfortunately, our experiments failed to find UMP-regulated ISG20 expression in cancer cells. We, therefore, further tested whether CD and m^6^_2_A could affect ISG20 expression and found that both promoted ISG20 expression at the protein and mRNA levels. CD is a natural active component of traditional Chinese medicine (TCM) fungus *cordyceps militaris*, which has anticancer properties (33–35). m^6^_2_A is a modified ribonucleoside in the tRNA of *mycobacterium bovis*, according to Bacille Calmette-Guérin (36). CD and m^6^_2_A are nucleoside derivatives that have been reported to inhibit the expression of CTSL, another SARS-CoV-2 receptor, in cancer cell lines (26). In addition, CD inhibited the expression of furin, another SARS-CoV-2 receptor, in several cancer cell lines (17). As CTSL inhibitors, both CD and m^6^_2_A can promote ISG20 upregulation. Considering ISG20 inhibits viral replication and/or degradation, CD and m^6^_2_A may play roles in preventing SARS-CoV-2 invasion and the severity of cancer.

Altogether, our study revealed the expression and distribution patterns of *ISG20* in virus/SARS-CoV-2 invasion inhibition on different tissues and organs, differential expression and methylation patterns, and the prognostic significance across several types of cancer. *ISG20* can play an important role in SARS-CoV-2 inhibition in certain types of cancer. Although future studies are needed for validation, our current study provides useful information to understand the current COVID-19 pandemic better. Moreover, small molecules from TCM or natural products may be used in the development of anti-SARS-CoV-2 drugs as well anticancer agents by upregulating ISG20 expression. Our study highlighted the value of targeting ISG20 as an alternative therapeutic strategy in combating cancer, SARS-CoV2, and other viral-caused diseases such as HAV, HBV, HCV, IAV, YFV, and BTV.

## Data availability statement

The original contributions presented in the study are included in the article/**Supplementary Material**. Further inquiries can be directed to the corresponding authors.

## Ethics statement

This study was reviewed and approved by The study was approved by the Ethical Committee of Southwest Medical University and The Affiliated Huaian No. 1 People’s Hospital of Nanjing Medical University. The patients/participants provided their written informed consent to participate in this study.

## Author contributions

JC, QT, JWF, ZL, XL, KG, LZ, JH, BZ, DL performed experimental studies, data acquisition, data analysis, and literature search. JJF collected and analyzed the data. JJF wrote and edited the manuscript. JJF, JC, DL revised the manuscript. All authors contributed to the article and approved the submitted version.

## Funding

This work was supported by the Foundation of Science and Technology Department of Sichuan Province (grant no. 2022NSFSC0737), the Foundation of Southwest Medical University (grant nos. 2021ZKMS004, 2021ZKQN109), in part by the Research Foundation of Luzhou City (grant no. 2021-SYF-37), and the National Natural Science Foundation of China (grant nos. 81672887 and 82073263).

## Acknowledgments

The authors thank all the people from the Research Center for Preclinical Medicine, Southwest Medical University.

## Conflict of interest

The authors declare that the research was conducted in the absence of any commercial or financial relationships that could be construed as a potential conflict of interest.

## Publisher’s note

All claims expressed in this article are solely those of the authors and do not necessarily represent those of their affiliated organizations, or those of the publisher, the editors and the reviewers. Any product that may be evaluated in this article, or claim that may be made by its manufacturer, is not guaranteed or endorsed by the publisher.
