# Supplementary material for: The regulation of ISG20 expression on SARS-CoV-2 infection in cancer patients and healthy individuals
Source: Front Immunol. 2022 Sep 13;13:958898. doi: 10.3389/fimmu.2022.958898 (PMC9513371; doi:10.3389/fimmu.2022.958898)
Supplement: Supplementary file 1 [file Presentation_1.pdf]

Supplementary Figures

Supplementary Figure 1

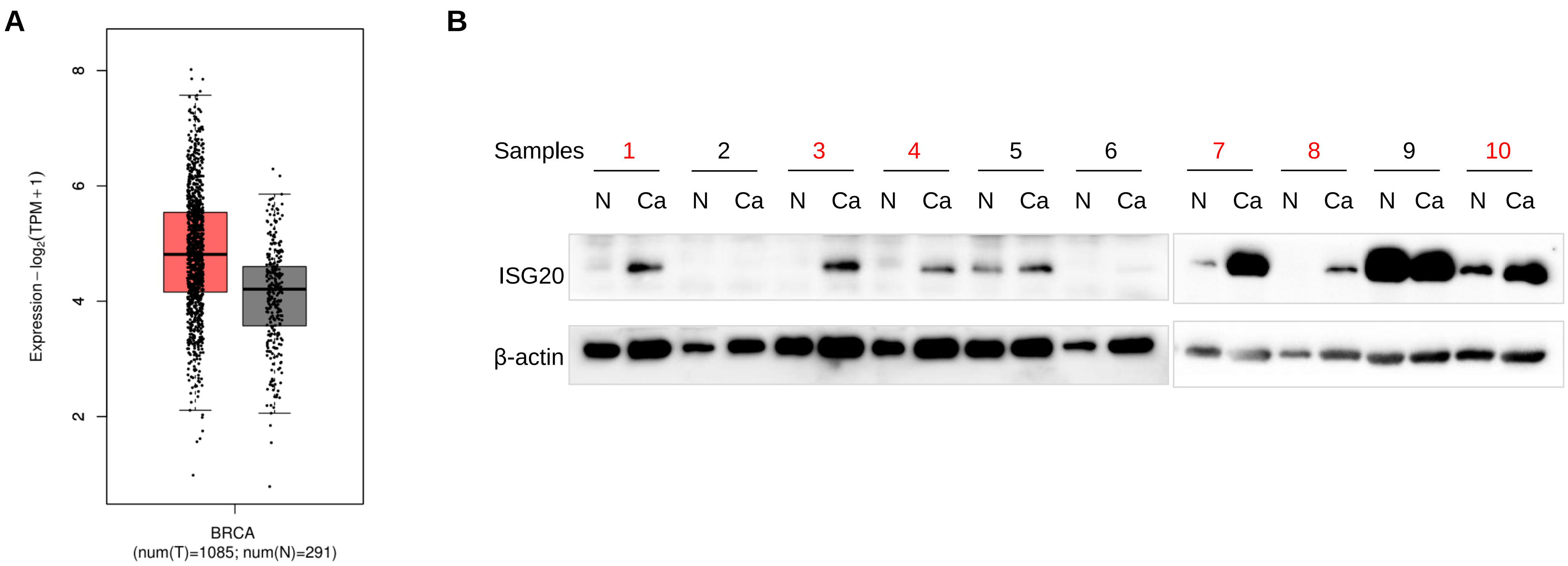

Supplementary Figure 1. The expression of ISG20 in the breast cancer tissues and in the respectively matched healthy tissues. A. The mRNA expression of ISG20 in the BRCA (breast invasive carcinoma) tissues and in the respectively matched healthy tissues from TCGA database. B. The protein expression of ISG20 in the breast cancer tissues and in the respectively matched healthy tissues from Chinese breast cancer patients. Red colors indicate ISG20 protein levels which were increased significantly when compared with the matched healthy tissues.

Supplementary Figure 2

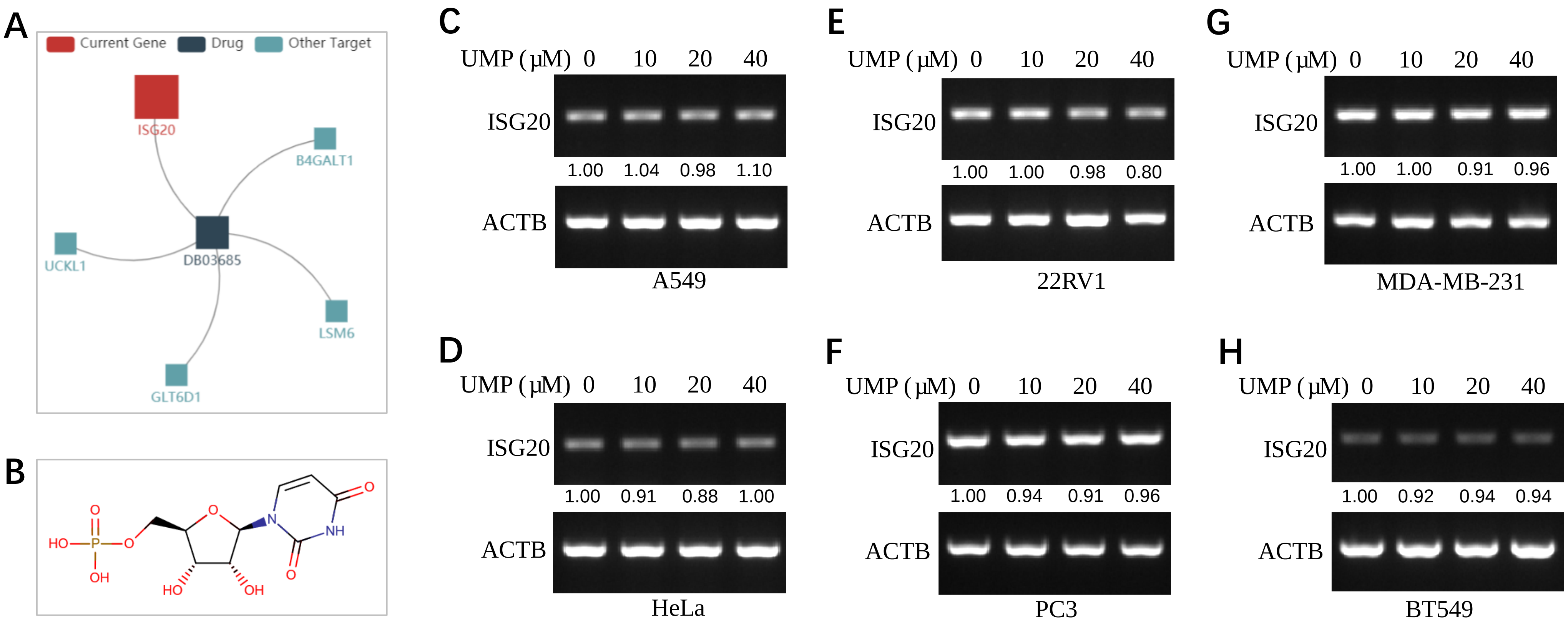

Supplementary Figure 2. Roles of uridine-5'-monophosphate (UMP) in ISG20 expression. A. Drugs targeting ISG20 were obtained from the DrugBank database. TISIDB: an integrated repository portal for tumor-immune system interactions (http://cis.hku.hk/TISIDB/browse.php?gene=ISG20). B. Structure of UMP. C-H. UMP increases ISG20 expression in the A549, HeLa, 22RV1, PC3, MDA-MB-231, and BT549 cancer cell lines.
